# Effect of Repeated Administration of ɣ-Valerolactone (GVL) and GHB in the Mouse: Neuroadaptive Changes of the GHB and GABAergic System

**DOI:** 10.3390/ph16091225

**Published:** 2023-08-30

**Authors:** Paolo Frisoni, Giorgia Corli, Sabrine Bilel, Micaela Tirri, Laura Camilla Gasparini, Letizia Alfieri, Margherita Neri, Fabio De-Giorgio, Matteo Marti

**Affiliations:** 1 Unit of Legal Medicine, AUSL of Ferrara, Via Arturo Cassoli 30, 44121 Ferrara, Italy; paolo.frisoni@ausl.fe.it; 2 Department of Translational Medicine, Section of Legal Medicine and LTTA Centre, University of Ferrara, 44121 Ferrara, Italy; giorgia.corli@unife.it (G.C.); sabrine.bilel@unife.it (S.B.); micaela.tirri@unife.it (M.T.); matteo.marti@unife.it (M.M.); 3 Department of Biomedical, Metabolic and Neural Sciences, Institute of Legal Medicine, University of Modena and Reggio Emilia, Via del Pozzo 71, 41124 Modena, Italy; laurac.gasparini@gmail.com; 4 Department of Medical Sciences, Section of Legal Medicine, University of Ferrara, Via Fossato di Mortara 70, 44121 Ferrara, Italy; letizia.alfieri@unife.it; 5 Department of Health Care Surveillance and Bioethics, Section of Legal Medicine, Università Cattolica del Sacro Cuore, 00168 Rome, Italy; fabio.degiorgio@unicatt.it; 6 Fondazione Policlinico Universitario A. Gemelli IRCCS, Largo F. Vito 1, 00168 Rome, Italy; 7 Collaborative Center for the Italian National Early Warning System, Department of Anti-Drug Policies, Presidency of the Council of Ministers, 00186 Rome, Italy

**Keywords:** GVL, GHV, GHB, GABAergic system, drug facilitated sexual assault, date-rape drug, new psychoactive substances, cardio-respiratory changes, sedative-hypnotics

## Abstract

Background: Gamma-hydroxybutyric acid (GHB) at low dosages has anxiolytic effects and promotes REM sleep and low-wave deep sleep. In the U.S., the legal form of GHB is prescribed to adults suffering from narcolepsy-associated cataplexy; the sodium salt of GHB is reserved for alcohol-addiction treatment. GHB is also a molecule of abuse and recreational use, it is a controlled substance in several countries, so gamma-valerolactone (GVL) has frequently been used as a legal substitute for it. GHB’s abuse profile is most likely attributable to its anxiolytic, hypnotic, and euphoric properties, as well as its widespread availability and inexpensive/low cost on the illicit market. Methods: Our study is focused on evaluating the potential effects on the mouse brain after repeated/prolonged administration of GHB and GVL at a pharmacologically active dose (100 mg/kg) through behavioral study and immunohistochemical analysis using the markers tetraspanin 17 (TSPAN17), aldehyde dehydrogenase 5 (ALDH5A1), Gamma-aminobutyric acid type A receptor (GABA-A), and Gamma-aminobutyric acid type B receptor (GABA-B). Results: Our findings revealed that prolonged administration of GHB and GVL at a pharmacologically active dose (100 mg/kg) can have effects on a component of the mouse brain, the intensity of which can be assessed using immunohistochemistry. The findings revealed that long-term GHB administration causes a significant plastic alteration of the GHB signaling system, with downregulation of the putative binding site (TSPAN17) and overexpression of ALDH5A1, especially in hippocampal neurons. Our findings further revealed that GABA-A and GABA-B receptors are downregulated in these brain locations, resulting in a greater decrease in GABA-B expression. Conclusions: The goal of this study, from the point of view of forensic pathology, is to provide a new methodological strategy for better understanding the properties of this controversial substance, which could help us better grasp the unknown mechanism underlying its abuse profile.

## 1. Introduction

Gamma-hydroxybutyric acid (GHB) is a short-chain fatty acid that is naturally present in the mammalian brain and has a neuromodulatory property at central GABAergic synapses, where it is synthesized from gamma-aminobutyric acid (GABA) in presynaptic neurons. GABA is the main inhibitory neurotransmitter in human brain cells, and it primarily reduces neuronal excitability throughout the nervous system [1]. GHB is an exogenous molecule, whose pharmacological form was first synthesized in 1874 by the reduction of succinyl chloride [2]. The first extended studies on the physiological effects of GHB were conducted in the 1960s; through human clinical trials, it was discovered that GHB is a structural analogue of GABA, active after oral administration, and could cross the blood–brain barrier, acting as a central nervous system depressant. Based on these findings, in 1963, Bessman and Fishbein discovered that GHB is not just a drug, but also an endogenous constituent of the mammalian brain and a metabolite of GABA [3].

Due to its interaction with the GABAergic system, this drug acts as a central nervous system depressant, and it was first employed as an intravenous anesthetic. High dosages can cause deep sedation with minimal effects on cardiovascular and respiratory depression without a significant interaction with liver and renal function. Despite this, GHB’s inefficient analgesic outcome led to its discontinuation in the anesthesiology field, and it was replaced with safer intravenous anesthetic drugs. The oral form of commercialized GHB became extremely popular as a dietary supplement in the 1990s, especially among athletes. Although it has never been demonstrated that GHB might improve athletes’ performance [4], it was thought that this substance had a slimming effect and could stimulate the secretion of growth hormone, increasing muscle mass. The use of GHB then spread to other social groups, allowing for the identification of the molecule’s abuse profile and recreational use. In humans, low dosages of GHB induce anxiolytic effects and promote REM sleep and low-wave deep sleep; in the U.S., the legal form of GHB (which was marketed under the proprietary name Xyrem^®^ and the generic name of “sodium oxybate”) is prescribed to adults suffering from narcolepsy-associated cataplexy. Its daily dose (4.5 g), taken before bedtime, is associated with a restorative effect on sleep and reduction of cataplexy episodes [5]. Similarly, the sodium salt of GHB is registered as Alcover^®^ in various European countries and its administration is reserved for alcohol-addiction treatment, as a support to abusers during detoxification and withdrawal. Compared with placebo treatment, the administration of sodium oxybate seems to be effective in maintaining abstinence, with less craving for alcohol and lower rates of relapse [6]. Higher dosages may cause a simultaneous combination of drug-related adverse effects, including euphoria and disinhibition caused by dopamine rush, as well as relaxant effects from GHB’s depressive action on the central nervous system. Thus, GHB’s abuse profile is most likely attributable to its anxiolytic, hypnotic, and euphoric properties, as well as its widespread availability and inexpensive/low cost on the illicit market. In the early 1990s, GHB was also labeled as “date-rape drug” and received mass media attention because of its implication in cases of drug-assisted sexual assault. This drug, which is typically odorless, colorless, and tasteless, is usually placed in drinks of unsuspecting victims, to incapacitate them and prevent resistance to sexual aggression. Due to the sedative effects and the retrograde amnesia induced by GHB, the victim may have little or no recollection of the events and many of them do not ever report the crime [7]. GHB is a controlled substance in several countries, so gamma-valerolactone (GVL) has frequently been used as a legal substitute for it. Unlike other GHB analogs, GVL is not metabolized to GHB but is processed by liver and plasma lactonase, which splits the lactone ring to gamma-hydroxyvaleric acid (GHV or 4-methyl-GHB). Therefore, after ingestion, the active compound is GHV, a homolog of GHB. Compared with GHB, the affinity of GHV for the GHB receptor is approximately two-fold lower than the endogenous substrate, as determined in rat brain preparations [7]: studies have shown that GHV has a relevant affinity for the GHB receptor in the brain, showing similar effects to GHB, even though it is less potent [8]. In vivo studies revealed that both GHB and GHV produce ataxia and catalepsy in a dose-dependent manner in rats [7,9]. Moreover, the gavage administration of GVL at a dose of 400 mg/kg significantly reduced the visual placing response in the first 4 h after intake and the effect disappeared after 5 h [10]. Intoxications with GVL, or its use as a “date rape drug”, are conceivable [11]. The published studies that have analyzed detection of GHV in human urine samples, report concentrations between 3 and 5.8 mg/L. GVL produces GHB-like effects, such as sedation, catalepsy, and ataxia, although larger doses of GHV are required to produce these effects [12]. Clinical studies on healthy human volunteers indicated the existence of a capacity-limited absorption of GHB, implying that the elimination mechanism of GHB becomes more relevant in situations of GHB intoxication at larger doses [13]. The clinical presentation of GHB intoxication [14] depends on the dose, route of administration, individual tolerance to depressant drugs, and co-administration of other drugs; indeed, correctly identifying GHB intoxication is a medical challenge because the signs and symptoms (i.e., feelings of relaxation, euphoria, confusion, hallucinations, nausea, somnolence, dizziness, etc.) are not specific [15]. Endogenous GHB has traditionally been thought as a neurotransmitter or neuromodulator in the mammalian brain [16]. Its neuronal synthesis in mammals follows at least two different pathways: the most important one, from a quantitative point of view, is represented by the conversion of GABA into succinic semialdehyde (SSA), and the subsequent reduction of SSA to GHB [17]. Endogenous GHB has been detected in all the investigated brain regions in different animal species: in the adult rat brain, for instance, GHB levels range from 0.4 μM in the frontal cortex, to 1.2 μM in the hippocampus, and 1.8 μM in the striatum. Its concentrations are highest in the human and monkey brains, reaching 11–25 μM in the striatum. Concerning the adult rat’s brain, the distribution of GHB high-affinity binding sites was studied by Maitre in 1996 through the use of [3H] GHB [18]. The maximal high-affinity binding was detected in different areas of the hippocampus; the cortex and the septum both had equivalent quantities of GHB binding sites, whereas the amygdala and the thalamus had intermediate levels, according to the study. Unlike these areas, the cerebellum and hypothalamus appeared to be devoid of high-affinity GHB binding sites under the conditions used to detect them. Other authors aimed to detect a GHB receptor-like protein in the rat brain using immunohistochemistry [19]. According to the study, the labeling was distributed in brain regions known to have high-affinity binding sites: cerebral cortex, hippocampus, and piriform cortex. The striatum was more lightly labeled, while the cerebellum appeared to be less immunoreactive, with positivity expressed especially at Purkinje cell dendritic trees. TSPAN17 is a probable GHB binding site, and the ALD5HA1 enzyme is involved in GHB metabolism, thus, we chose those targets. We analyzed different brain areas of the murine central nervous system (CNS) (cortex, striatum, hippocampus, and cerebellum) that are known to be involved in GHB pharmacological effects and controlled by GABAergic transmission. Indeed, GHB shows both high- and low-affinity binding targets in the CNS [20]; the most well-established target for GHB is the GABA-B receptor, at which GHB displays weak affinity and a partial agonist action with millimolar potency [21]. The major pharmacological and behavioral effects of exogenous GHB are mediated via GABA-B receptors, including properties of addiction and tolerance. Indirect effects may also occur if GHB is converted to GABA, which, in turn, can activate GABA-B (or GABA-A) receptors [20]. The study of Schuler et al. on mice lacking functional GABA-B receptors showed that all pharmacological and behavioral effects induced by GHB administration (hypothermia, hypolocomotion, increase in striatal dopamine synthesis, EEG abnormalities) were mediated by GABA-B receptor activation [22].

The aim of our study is to evaluate the potential effects on the component of mouse brain after repeated/prolonged administration of GHB and GVL at a pharmacologically active dose devoid of sedative effects and stimulating locomotion in mice (100 mg/kg; [21]) through behavioral study and immunohistochemical analysis using the markers TSPAN17, ALDH5A1, GABA-A, and GABA-B.

## 2. Results

### 2.1. Motor Activity Assessment

**Figure 1:**
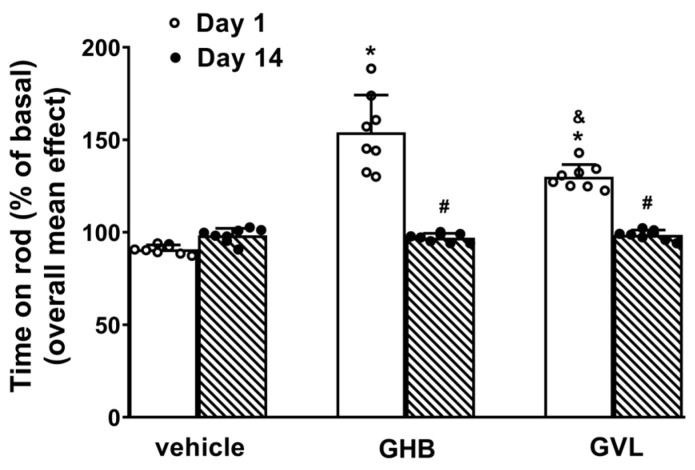
Effect of acute (Day1) and repeated (Day 14) gavage administration (100 mg/kg) of GHB (Group 1) and GVL (Group 2) on the accelerod test in mice. Data are expressed as a percentage of baseline and are reported as the mean effects during the 5 h observation. Data represent the mean ± SEM of 8 determinations for each treatment. Statistical analysis was performed by one-way ANOVA followed by the Tukey’s test for multiple comparisons of each group. * *p* < 0.05 GHB (Group 1) or GVL (Group 2) vs. vehicle (Group 3) at Day 1; ^&^ *p* < 0.05 GVL (Group 2) vs. GHB (Group 1) at Day 1; ^#^ *p* < 0.05 Day 14 vs. Day 1.

Alterations of motor activity induced by GHB and GVL were measured using the accelerod tests [23]. Acute (Day 1) and repeated (Day 14) administration of the vehicle did not change the motor performance of mice in the accelerod test (Figure 1). Acute gavage administration (Day 1) of GHB and GVL at 100 mg/kg induced an increase of overall motor performance (Figure 1: F(5,38) = 51.38, *p* < 0.0001), and the overall effect induced by GHB was greater in respect to that caused by GVL administration (Figure 1, *p* = 0.0128). The facilitatory effect of GHB and GVL was lost after repeated administration (Day 14) of GHB and GVL (Figure 1).

### 2.2. Immunohistochemistry

#### 2.2.1. TSPAN17

**Figure 2:**
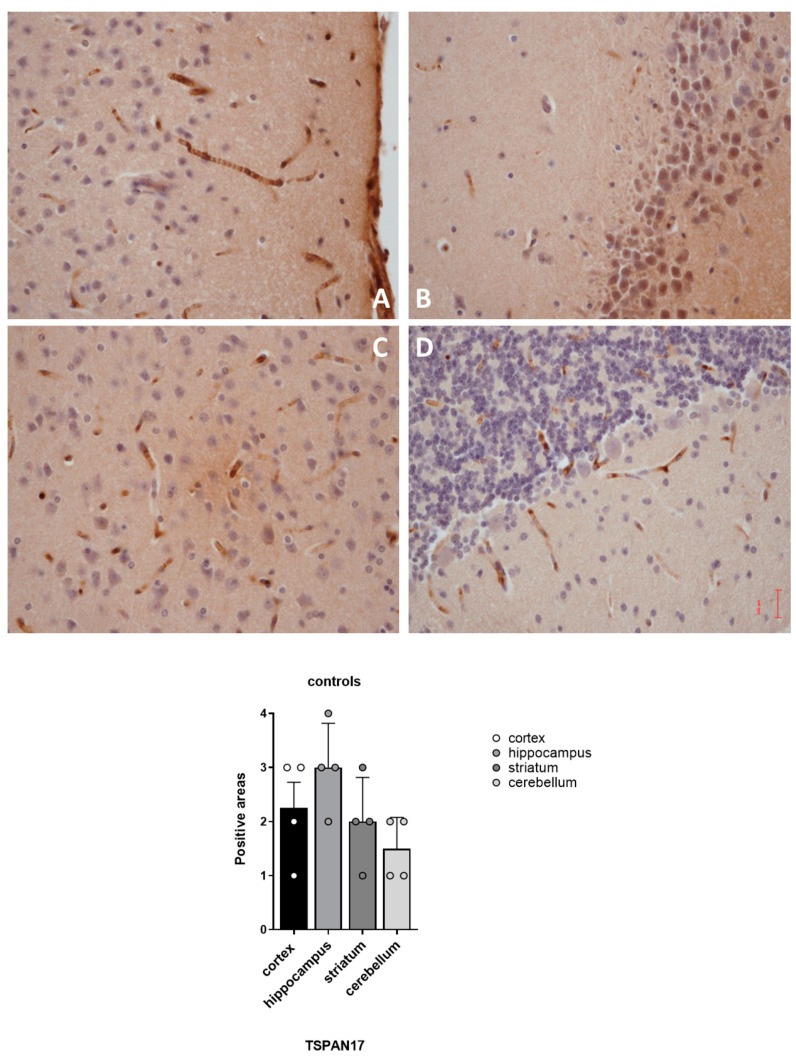
Distribution of TSPAN17 positive areas in the brain of vehicle-treated mice (Group 3). (**A**) cortex sample; (**B**) hippocampus sample; (**C**) striatum sample; (**D**) cerebellum sample.

The spatial distribution of TSPAN17 positivity in non-treated mice seems to agree with the previous studies, that show the localization of a GHB receptor-like protein in the rat brain [19]. Our study revealed that the labeling was distributed in brain regions known to express high-affinity binding sites, namely, the cerebral cortex, hippocampus and piriform cortex; the striatum was more lightly labeled while the cerebellum appeared to be less immunoreactive, with positivity expressed especially at Purkinje cell dendritic trees [1]. The semi-quantitative evaluation of positive areas (Figure 2) shows that the highest concentration was detectable in the hippocampus, then in the cortex and striatum, with the lowest level of positive area in the cerebellum (Figure 2). This agreed with earlier evidence regarding the regional distribution of high affinity GHB binding sites in rodents. The TPSAN17 positivity observed in our evaluation can provide further evidence for the hypothesis that TSPAN17 could be a component of the GHB’s receptors system.

**Figure 3:**
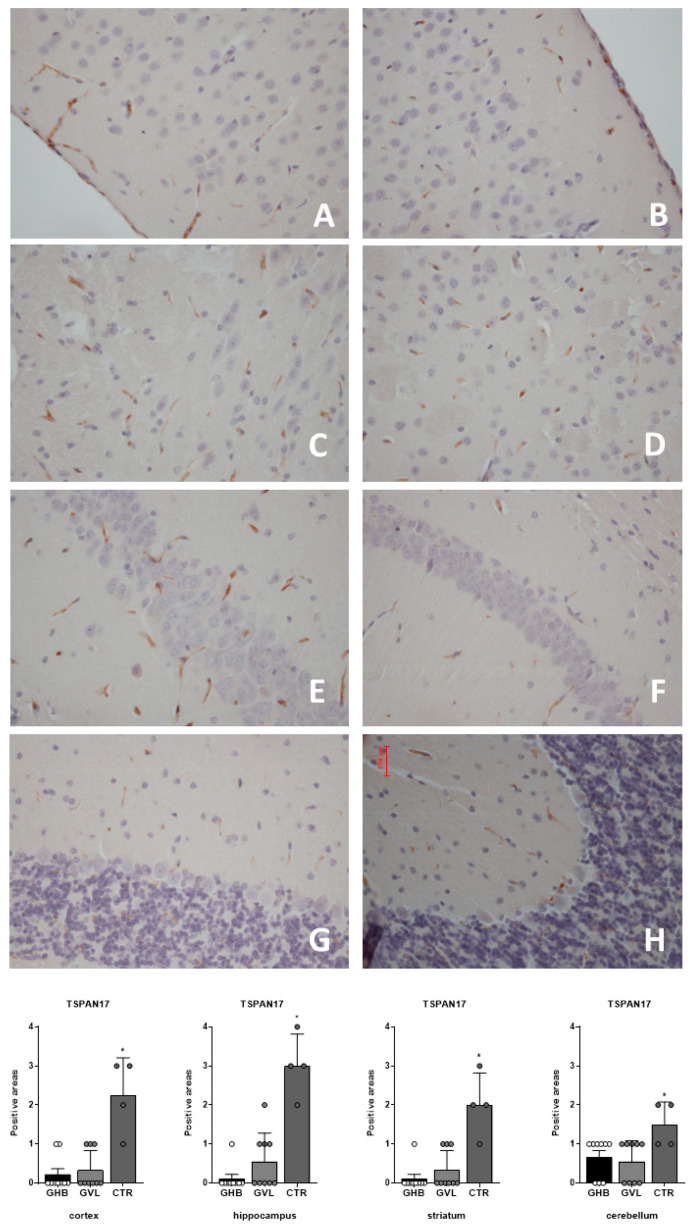
Distribution of TSPAN17 weak positive areas in the brain of treated mice. Data plot of Group1 (GHB) is shown as white circles, Group 2 (GVL) is shown as light grey circles, and Group 3 (controls) is shown as dark grey circles. (**A**) Group 1 cortex; (**B**) Group 2 cortex; (**C**) Group 1 hippocampus; (**D**) Group 2 hippocampus; (**E**) Group 1 striatum; (**F**) Group 2 striatum; (**G**) Group 1 cerebellum; (**H**) Group 2 cerebellum. Statistical analysis result shows a significant hypo-expression of TSPAN17 in treated mice (GHB: Group 1; and GVL: Group 2) vs. control group (vehicle: Group 3) * *p* < 0.05.

The evaluation of the positivity of TSPAN17 (Figure 3) in treated (GHB: Group 1 and GVL: Group 2) vs. control (vehicle: Group 3) groups has shown a common pattern of response in different brain regions: in all the samples collected from the cortex (F_(2,19)_ = 19.26, *p* < 0.0001), hippocampus (F_(2,19)_ = 32.28, *p* < 0.0001), striatum (F_(2,19)_ = 20.53, *p* < 0.0001), and cerebellum (F_(2,19)_ = 4.805, *p* = 0205), there was evidence of a remarkable decrease of TSPAN17 immunoreactivity in treated mice vs. control animals, with a maximum decrease in the hippocampus and minimal decrease in the cerebellum (Figure 3). No difference was noticed between the mice treated with GHB and GVL.

#### 2.2.2. ALDH5A1

**Figure 4:**
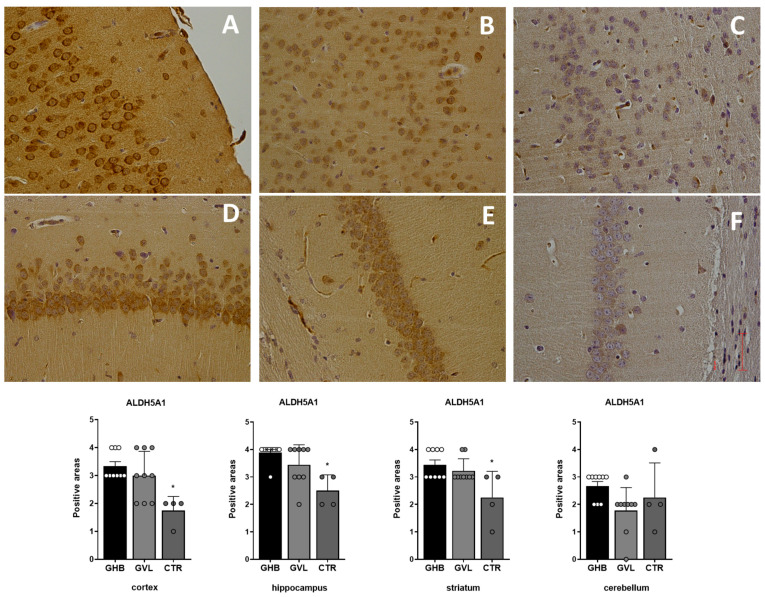
Distribution of ALDH5A1 intense positive areas in the brain of treated mice (Group1 and Group 2) and a very weak expression in Group 3. Data plot of Group1 (GHB) is shown as white circles, Group 2 (GVL) is shown as light grey circles, and Group 3 (controls) is shown as dark grey circles. (**A**): Group 1 cortex; (**B**): Group 2 cortex; (**C**): Group 3 cortex; (**D**): Group 1 hippocampus; (**E**): Group 2 hippocampus; (**F**): Group 3 hippocampus. Statistical analysis result shows a significant overexpression of ALDH5A1 in treated mice (GHB: Group 1 and GVL: Group 2) vs. control group (vehicle: Group 3) * *p* < 0.05.

The evaluation of ALDH5A1 positivity in treated vs. control mice revealed a consistent pattern of response in the samples collected from the cortex (F_(2,19)_ = 7.674, *p* = 0.0036), hippocampus (F_(2,19)_ = 8.307, *p* = 0.0101), and striatum (F_(2,19)_ = 5.913, *p* = 0.0026). There was a widespread increase of ALDH5A1 binding in treated mice vs. control mice, especially in hippocampal and cortex samples (Figure 4). Statistical analysis showed a significant hypo-expression of ALDH5A1 in vehicle cases (Group 3) compared with those observed in both GHB- and GVL-treated mice (Group 1 and Group 2).

#### 2.2.3. GABA-A and GABA-B

**Figure 5:**
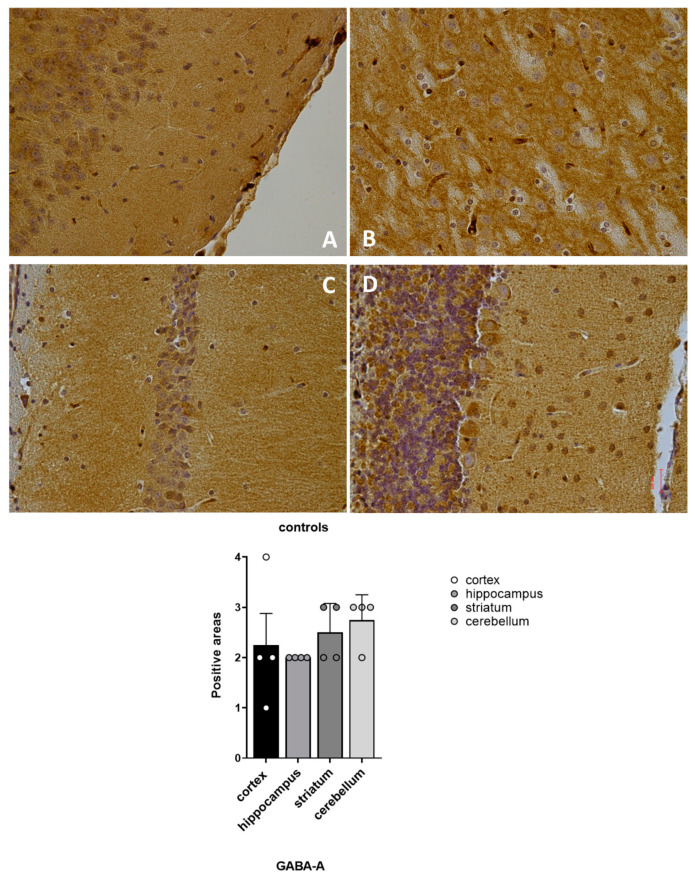
Distribution of GABA-A positive areas in CNS of non-treated mice Group 3. (**A**) cortex sample; (**B**) hippocampus sample; (**C**) striatum sample; (**D**) cerebellum sample.

**Figure 6:**
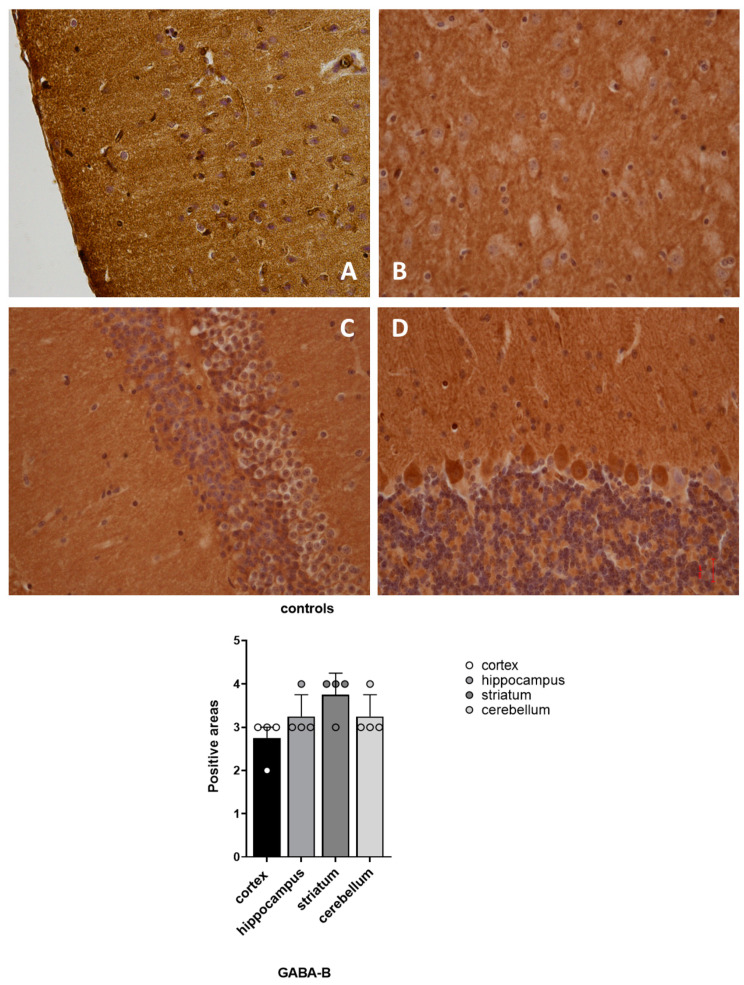
Distribution of GABA-B positive areas in CNS of non-treated mice Group 3. (**A**) cortex sample; (**B**) hippocampus sample; (**C**) striatum sample; (**D**) cerebellum sample.

Our results showed that GABA-A and GABA-B receptors were expressed in all of the four examined brain regions of the vehicle-treated mice (Group 3), with a spatial distribution that matched the outlined pattern (Figure 5 and Figure 6).

**Figure 7:**
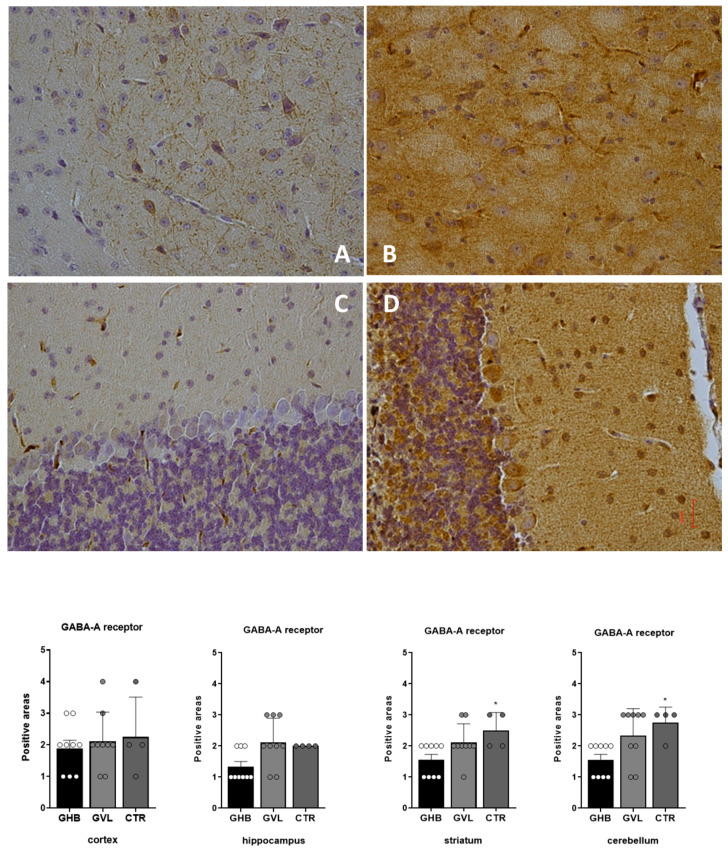
Distribution of GABA-A immunoreactivity in the brain areas of non-treated mice (Group 3) and a decreased expression in mice treated with GHB/GVL (Groups 1 and 2). Data plot of Group1 (GHB) is shown as white circles, Group 2 (GVL) is shown as light grey circles, and Group 3 (controls) is shown as dark grey circles. (**A**) Group 2 striatum; (**B**) Group 3 striatum; (**C**) Group 1 cerebellum; (**D**) Group 3 cerebellum. Statistical analysis result shows a significantly reduced expression of GABA-A in treated mice (GHB: Group 1 and GVL: Group 2) vs. control group (vehicle: Group 3) * *p* < 0.05.

The evaluation of GABA-A receptor positivity in GHB/GVL-treated mice (Groups 1 and 2) vs. control mice (Group 3), has shown a dissimilar pattern of response in different brain regions: in the four areas collected, a decrease of GABA-A immunoreactivity in treated mice vs. control animals (Group 1 and Group 2 vs. Group 3) was notable and statistically significant in the striatum (F_(2,19)_ = 4.420, *p* = 0.0265) and cerebellum (F_(2,19)_ = 5.131, *p* = 0.0165) samples, and not significant in the cortex and hippocampus (Figure 7).

**Figure 8:**
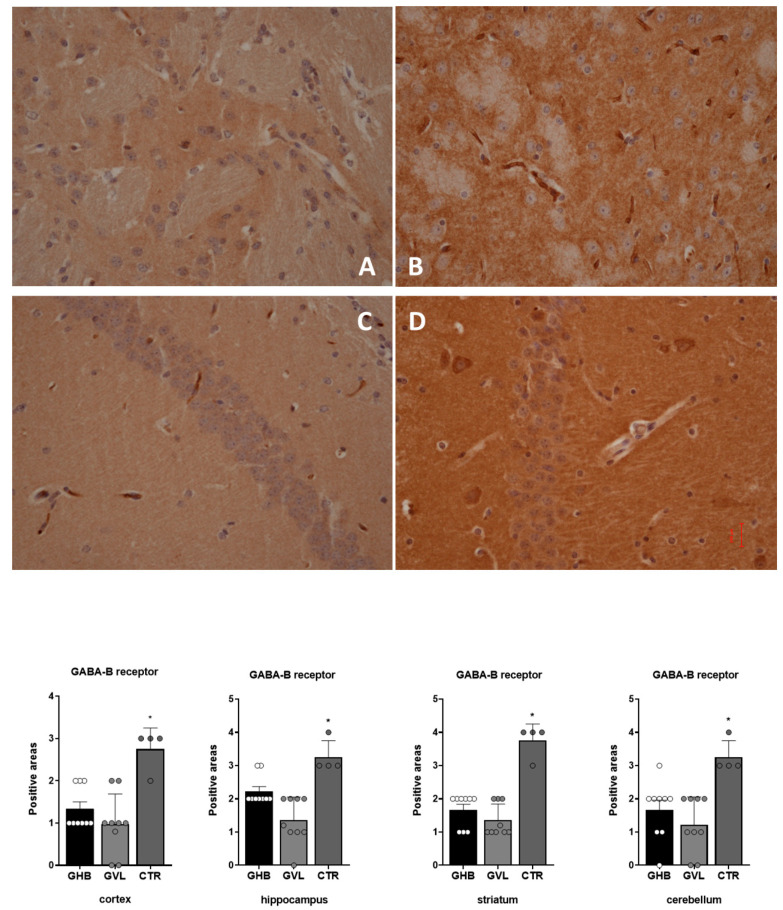
Distribution of GABA-B immunoreactivity in brain areas of non-treated mice (Group 3) and a decreased expression in mice treated with GHB/GVL (Groups 1 and 2). Data plot of Group1 (GHB) is shown as white circles, Group 2 (GVL) is shown as light grey circles, and Group 3 (controls) is shown as dark grey circles. (**A**) Group 1 striatum; (**B**) Group 3 striatum; (**C**) Group 2 hippocampus; (**D**) Group 3 hippocampus. Statistical analysis result shows a significantly reduced expression of GABA-B in treated mice (GHB: Group 1 and GVL: Group 2) vs. control group (vehicle: Group 3) * *p* < 0.05.

The evaluation of GABA-B receptor positivity in GHB/GVL-treated mice (Groups 1 and 2) vs. control mice (Group 3) revealed a common pattern of response in the four different brain regions; there was evidence of a decrease in GABA-B immunoreactivity in treated mice vs. control mice in all samples collected from the cortex (F_(2,19)_ = 12.44, *p* = 0.0004), hippocampus (F_(2,19)_ = 34.39, *p* < 0.0001), striatum (F_(2,19)_ = 15.86, *p* < 0.0001), and cerebellum (F_(2,19)_ = 8.923, *p* = 0.0019), with the smallest decrease in the hippocampus and the largest decrease in the striatum (Figure 8).

Concerning the results obtained from the evaluation of GABA-A and GABA-B receptor-immunoreactivity in GHB-treated mice, a known effect of chronic GHB exposure is the desensitization of the GABAergic system, which reduces the capacity of neurotransmitter release [24]. Our findings appeared to be consistent with previous research, demonstrating a downregulation of the entire GABAergic system, even with a greater emphasis on GABA-B receptor activity. 

## 3. Discussion

GHB is rapidly absorbed [25] and metabolized (average half-life, 40–60 min after a single oral dose; detection windows, 4–5 h in blood or serum) [12]. The current knowledge of its pharmacokinetic properties derives from controlled human dosing trials. Effects on motor performance and asphyxia were detecTable 15–30 min after oral consumption of a dose of GHB, with subjective effects (peak between 1 and 1.5 h after drug administration) reported as a mixed sedative–stimulant pattern, and volunteers experiencing euphoria, disinhibition, and drowsiness. It is worthy to note that even small increases in the oral administration dose can result in non-proportional increases in the subjective effects experienced by users: after each dose, the plasmatic concentration of GHB showed a biphasic decay phase, with an initial rapid decline followed by a convex concentration time profile, which became more prominent as the dose increased. Clinical studies on healthy human volunteers indicated the existence of a capacity-limited absorption of GHB, implying that the elimination mechanism of GHB becomes more relevant in the case of intoxication caused by larger doses of GHB [13].

Due to GHB’s narrow therapeutic window and peculiar pharmacodynamic properties, there is a small margin of safety between a recreational dose and a fatal one. Furthermore, the risk of an adverse event occurring after ingesting GHB for recreational purposes is linked not only to the drug’s steep dose–response curve, but also to the variability of its clinical effects. Moreover, the mechanism of postmortem production of the molecule is also a problem in terms of GHB analysis. Although the process of post-mortem GHB synthesis has not been clarified, endogenous post-mortem concentrations were found to be significantly higher than those detected in ante-mortem samples [26].

In our study, we investigated the neuroplastic alterations in the GABA-B and GABA-A receptors (since GHB action involves GABAergic transmission) and conducted an immunohistochemical study using antibodies directed against the TSPAN17 site, the ALDH5A1 enzyme, the GABA-B receptor 1, and GABA-A receptor alpha 1 subunit. TSPAN17 is a probable GHB binding site, and the ALD5HA1 enzyme is implicated in GHB metabolism. We studied different brain areas (cortex, striatum, hippocampus and cerebellum) that are known to be involved in GHB pharmacological effects and controlled by GABAergic transmission. 

We administered a dose of GHB and GVL (100 mg/kg) devoid of sedative effects and that acutely stimulate the overall motor performance of mice [21]. The motor facilitation induced by this low dose (100 mg/kg) of GHB and possibly GVL could be due to the activation of the GHB receptors, which have been linked to an inhibition of GABAergic interneurons and consequent increase in dopamine levels [20]. In humans, it can be linked to a state of euphoria and disinhibition.

Interestingly, the facilitatory effect of GHB and GVL was lost after repeated administration (Day 14) of GHB and GVL, suggesting that tolerance mechanisms come into play. Immunohistochemical results supported the appearance of pharmacodynamics (downregulation of GABA-A, GABA-B and TSPAN17) and pharmacokinetic (upregulation of ALDH5A1 enzyme) adaptations that can cause the loss of effect of the two compounds. From a translational point of view, this implies that the subject must increase the dose of GHB and/or GVL to obtain the desired effects.

Regarding the result of our study, the spatial distribution of TSPAN17 positivity in non-treated mice seems to agree with the previous data reported. The semi-quantitative evaluation of positive areas showed that the highest concentration was detectable in the hippocampus, then in the cortex and striatum, with the lowest level of positive area in the cerebellum. The spatial concordance between earlier evidence regarding the regional distribution of high-affinity GHB binding sites in rodents and the finding of TPSAN17 positivity observed in our evaluation can provide further support to the hypothesis that TSPAN17 could be a component of the GHB receptor system. 

These findings agree with the results of our analysis, which demonstrated moderate–high GABA-B immunoreactivity in the cortex, hippocampus, and cerebellum; nevertheless, in contrast with the results described above, we found substantial binding even in striatal sample collection.

Our semi-quantitative assessment of positive GABA-A areas in non-treated mice appears to correlate with previously published results, demonstrating the presence of GABA-A receptors in all examined brain regions, with a spatial distribution that matches the outlined pattern.

The considerable decrease in TSPAN17 binding in GHB/GVL treated mice might be an expression of the process of GHB receptor downregulation, which could be interpreted as a mechanism of adaptation to chronic GHB treatment. The significant decrease in TSPAN17 expression in the hippocampal area is likely due to the high concentration of GHB binding sites physiologically present in this area, making this brain region more susceptible to receptor downregulation induced by supraphysiological doses of GHB; on the other hand, the cerebellum, a brain region with lower expression, has shown a minor degree of downregulation.

In this regard, the hippocampus is a brain region implicated in learning and memory formation [27]; previous research on this neuronal area has revealed that GHB can impair long-term potentiation by acting on the GABA-B receptor, resulting in reduced learning and memory retention [28]. Our data suggest that this effect could be magnified by the simultaneous downregulation of the GHB receptor system.

The evaluation of GABA-B receptor positivity in GHB/GVL-treated vs. control mice revealed a common pattern of response in different brain regions: there is evidence of a decrease in GABA-B binding in treated mice vs. control mice in all collected samples, with the smallest decrease in the hippocampus and the largest decrease in the striatum. The evaluation of GABA-A receptor positivity in treated vs. control mice, showed a dissimilar pattern of response in different brain regions: in the samples collected, a decrease of GABA-A binding in treated mice vs. control animals was notable, with particular reference to the striatum and cerebellum samples. Our results demonstrated a downregulation of the entire GABA system and appear to be consistent with previous research. The evaluation of ALDH5A1 positivity in treated vs. control mice revealed a consistent pattern of response in different brain regions: in the samples collected, there was a widespread increase in ALDH5A1 binding in treated mice vs. control mice, especially in hippocampal and cortex samples. The enzymatic increase in succinic semialdehyde dehydrogenase activity, shown as an overexpression of ALDH5A1 in all of the studied tissue samples, may represent an adaptive response to the global downregulation of GHB receptor caused by the chronic exposure to the drug, reflecting GHB’s ability to alter neuronal plasticity.

Our findings revealed that prolonged administration of GHB and GVL at a pharmacologically active dose (100 mg/kg) can have effects on the mouse brain, the intensity of which can be assessed using immunohistochemistry. The findings revealed that long-term GHB administration causes a significant plastic alteration of the GHB signaling system, with downregulation of the putative binding site (TSPAN17) and overexpression of ALDH5A1, especially in hippocampal neurons. Our findings further revealed that GABA-A and GABA-B receptors are downregulated in these brain locations, resulting in a greater decrease in GABA-B expression. In addition, our results showed that, in terms of magnitude of neural plasticity induction, GHB is more effective than GVL, as evidenced by prior research [9].

Chronic use of GHB causes a downregulation of the GABAergic system, as observed in our study. The activation of the mesolimbic dopamine system, thought to be involved in the induction of drug use, could hypothetically explain the increased reward addictive process that accentuates craving and GHB-seeking behavior, providing a possible explanation for GHB strong abuse potential [17].

## 4. Materials and Methods

### 4.1. Animals

Male ICR (CD-1^®^) mice weighing 30–35 g (Centralized Preclinical Research Laboratory, University of Ferrara, Ferrara, Italy) were group-housed (5 mice per cage; floor area per animal was 80 cm^2^; minimum enclosure height was 12 cm), exposed to a 12:12 h light–dark cycle (light period from 6:30 a.m. to 6:30 p.m.) at a temperature of 20–22 °C and humidity of 45–55%. Water and food (diet 4RF25 GLP; Mucedola, Settimo Milanese, Milan, Italy) were provided ad libitum. The experimental protocols performed in the present study were in accordance with the U.K. Animals (Scientific Procedures) Act of 1986 and associated guidelines, and the new European Communities Council Directive of September 2010 (2010/63/EU). These were approved by the Italian Ministry of Health (license no. 223/2021-PR and extension CBCC2.46.EXT.21) and by the Animal Welfare Body of the University of Ferrara. According to the ARRIVE guidelines, all possible efforts were undertaken to minimize the number of animals used, and the animals’ pain and discomfort.

### 4.2. Drug Preparation and Animal Dose Determination

GVL was purchased from Sigma Aldrich, Milano, Italy, and GHB (sodium salt) was purchased from LCG standards S.r.l. Both compounds were diluted (GVL) or dissolved (GHB) in saline (0.9% NaCl) solution that was also used as a vehicle and administered orally using gavage needles at a volume of 4 μL/g. The dose of GHB and GVL (100 mg/kg) was chosen based on our previous study [10,29].

### 4.3. Motor Activity Assessment

Alterations of motor activity induced by GHB and GVL were measured using the accelerod test [23]. In the accelerod test, animals were placed on a rotating cylinder that increases velocity automatically in a constant manner (0–60 rotations/min in 5 min). The time spent on the cylinder was measured. The accelerod test was performed at 0, 40, 65, 95, 150, 210, 270, and 330 min post-administration, and the overall mean effect was calculated. All experiments were performed between 8:30 a.m. and 2:00 p.m. Experiments were conducted blindly by trained observers working in pairs [23].

### 4.4. Laboratory Protocol

A total of 22 mice were employed in the immunohistochemistry study and were randomly divided into three groups. Group 1 included nine GHB-treated mice (denoted as 1 to 9); all mice belonging to this group were treated with 100 mg/kg of GHB, which was administered via oral gavage every day at the same time for a total of 14 days. Group 2 included nine GVL-treated mice (denoted as 10 to 18); the mice belonging to this group were treated with 100 mg/kg of GVL, which was administered via oral gavage every day at the same time for a total of 14 days. Finally, Group 3 (“control group”) included four mice that were neither treated with GHB nor with GVL (denoted as C1, C2, C3, C4); these mice were administered via oral gavage every day at the same time for a total of 14 days with saline (0.9% NaCl) solution that was also used as a vehicle. 

### 4.5. Immunohistochemistry Analysis

The mice brains were removed and fixed in 10% buffered formalin for 48 h. Each fixed brain was then dissected into four specimens named A, B, C, and D proceeding, respectively, from the anterior to the posterior cerebral regions. Each of these sections was then processed for paraffin inclusion. All samples of the four areas of brain (cortex, striatum, hippocampus and cerebellum) were evaluated through immunohistochemical investigations for TSPAN17, ALDH5A1, GABA-A and GABA-B. For each sample, sections of 4 μm thickness were cut; after the hydration, the slices were pretreated for antigen retrieval and then incubated with primary antibody according to the dilution indicated in Table 1. The detection system (CTS005 HRP-DAB system R&D kit, R&D systems, Inc., Minneapolis, MN, USA) was a refined avidin–biotin system in which a biotinylated secondary antibody reacted with several peroxidise-conjugated streptavidin molecules. The positive reaction was visualized by 3,3′-diaminobenzidine (DAB) peroxidation, according to standard methods. There was presence of non-specific markings due to the avidin–biotin system. Hence, we carried out tests using a polymer system (BioCare Goat-on-rodent HRP-Polymer, BioCare Medical, Pacheco, CA, USA) to obtain the markings of the same areas. Sections were counterstained with hematoxylin, then dehydrated, cover-slipped and observed in a Nikon Eclipse E600 microscope (Nikon, Tokyo, Japan).

TSPAN17, ALDH5A1 (D-3), GABA-A Receptor alpha 1 and GABA-B Receptor 1 immunoreactivity were subjected to a semi-quantitative evaluation according to previous studies, because the type of reaction is better evaluated using a semiquantitative method [30]. Each slide was evaluated by 2 different investigators at × 40 magnification. The intensity of immunopositivity was assessed semi-quantitatively and expressed on a scale of 0–5 as follows: − , no immunoreactivity (0%); + /–, basal immunopositivity (5%); + , mild immunopositivity (10%); + + , isolated immunopositivity (33%); + + + , diffuse immunopositivity (66%), and + + + + , widespread immunopositivity (>90%). In cases of divergent scores, a third investigator decided the final score.

### 4.6. Data and Statistical Analysis

In motor response experiments (accelerod test) data are expressed as overall mean effect of percentage of basal value. The statistical analysis of the motor effects of the individual substances were performed using a one-way analysis of variance (ANOVA), followed by Tukey’s post-hoc test for multiple comparisons. The statistical analysis of immunohistochemical experiments were checked for normality using Bartlett’s test and analyzed by one-way analysis of variance (ANOVA), followed by Tukey’s post-hoc test. For all tests, a *p* value < 0.05 was considered statistically significant. Results were expressed as means ± mean standard deviation. Data were analyzed using the GraphPad Prism 8 software for Windows (San Diego, CA, USA).

## 5. Conclusions

The disappearance of behavioral effects and the results of immunohistochemistry highlighting downregulation of the putative binding site (TSPAN17) and overexpression of ALDH5A1, a downregulation of GABA-A and GABA-B receptors, state the perfect agreement between the two analyses. In conclusion, our study could point to a new methodological strategy for better understanding the properties of this controversial substance, which could help us better grasp the unknown mechanism underlying its abuse profile in subjects and could also be helpful in the analysis of victims in cases where GHB is used as a “date rape drug”.

## Figures and Tables

**Table 1:** Antibody dilutions.

Antibody	Concentration of Primary Antibody
ALDH5A1 (D-3) (sc-390754; Santa Cruz Biotechnology, Santa Cruz, California, U.S.A.)	1:500
TSPAN17 (ab180601; Abcam, Cambridge, U.K.)	1:200
GABA-A Receptor alpha 1 (ab33299; Abcam, Cambridge, U.K.)	1:500
GABA B Receptor 1 (ab55051; Abcam, Cambridge, U.K.)	1:2000

## Data Availability

Data is contained within the article.

## References

[1] Maitre M. (1997). The Gamma-Hydroxybutyrate Signalling System in Brain: Organization and Functional Implications. Prog. Neurobiol..

[2] Busardò F.P., Jones A.W. (2015). GHB Pharmacology and Toxicology: Acute Intoxication, Concentrations in Blood and Urine in Forensic Cases and Treatment of the Withdrawal Syndrome. Curr. Neuropharmacol..

[3] Fishbein W.N., Bessman S.P. (1964). Gamma-Hydroxybutyrate in Mammalian Brain. Reversible Oxidation by Lactic Dehydrogenase. J. Biol. Chem..

[4] Gonzalez A., Nutt D.J. (2005). Gamma Hydroxy Butyrate Abuse and Dependency. J. Psychopharmacol..

[5] Boscolo-Berto R., Viel G., Montagnese S., Raduazzo D.I., Ferrara S.D., Dauvilliers Y. (2012). Narcolepsy and Effectiveness of Gamma-Hydroxybutyrate (GHB): A Systematic Review and Meta-Analysis of Randomized Controlled Trials. Sleep Med. Rev..

[6] Marinetti L., Montgomery M.A. (2010). The Use of GHB to Facilitate Sexual Assault. Forensic Sci. Rev..

[7] Carter L.P., Chen W., Wu H., Mehta A.K., Hernandez R.J., Ticku M.K., Coop A., Koek W., France C.P. (2005). Comparison of the Behavioral Effects of Gamma-Hydroxybutyric Acid (GHB) and Its 4-Methyl-Substituted Analog, Gamma-Hydroxyvaleric Acid (GHV). Drug Alcohol Depend..

[8] Johansen S.S., Windberg C.N. (2011). Simultaneous Determination of γ-Hydroxybutyrate (GHB) and Its Analogues (GBL, 1.4-BD, GVL) in Whole Blood and Urine by Liquid Chromatography Coupled to Tandem Mass Spectrometry. J. Anal. Toxicol..

[9] Marinetti L.J., Leavell B.J., Jones C.M., Hepler B.R., Isenschmid D.S., Commissaris R.L. (2012). Gamma Butyrolactone (GBL) and Gamma Valerolactone (GVL): Similarities and Differences in Their Effects on the Acoustic Startle Reflex and the Conditioned Enhancement of Startle in the Rat. Pharmacol. Biochem. Behav..

[10] Camuto C., Arfè R., Tirri M., de la Torre X., Mazzarino M., Marti M., De-Giorgio F., Botrè F. (2022). Urinary Excretion and Effects on Visual Placing Response in Mice of Gamma-Valero-Lactone, an Alternative to Gamma-hydroxy-Butyrate for Drug-Facilitated Sexual Assault. Emerg. Trends Drugs Addict. Health.

[11] Andresen-Streichert H., Jungen H., Gehl A., Müller A., Iwersen-Bergmann S. (2013). Uptake of Gamma-Valerolactone--Detection of Gamma-Hydroxyvaleric Acid in Human Urine Samples. J. Anal. Toxicol..

[12] Borgen L.A., Okerholm R.A., Lai A., Scharf M.B. (2004). The Pharmacokinetics of Sodium Oxybate Oral Solution Following Acute and Chronic Administration to Narcoleptic Patients. J. Clin. Pharmacol..

[13] World Health Organization (2012). WHO Expert Committee on Drug Dependence.

[14] Madah-Amiri D., Myrmel L., Brattebø G. (2017). Intoxication with GHB/GBL: Characteristics and Trends from Ambulance-Attended Overdoses. Scand. J. Trauma Resusc. Emerg. Med..

[15] Miró Ò., Galicia M., Dargan P., Dines A.M., Giraudon I., Heyerdahl F., Hovda K.E., Yates C., Wood D.M., Liakoni E. (2017). Intoxication by Gamma Hydroxybutyrate and Related Analogues: Clinical Characteristics and Comparison between Pure Intoxication and That Combined with Other Substances of Abuse. Toxicol. Lett..

[16] Wong C.G.T., Chan K.F.Y., Gibson K.M., Snead O.C. (2004). Gamma-Hydroxybutyric Acid: Neurobiology and Toxicology of a Recreational Drug. Toxicol. Rev..

[17] Kamal R.M., van Noorden M.S., Franzek E., Dijkstra B.A.G., Loonen A.J.M., De Jong C.A.J. (2016). The Neurobiological Mechanisms of Gamma-Hydroxybutyrate Dependence and Withdrawal and Their Clinical Relevance: A Review. Neuropsychobiology.

[18] Cash C.D., Hechler V., Mersel M., Maitre M. (1996). Kinetic Characterisation and Solubilisation of Gamma-Hydroxybutyrate Receptors from Rat Brain. Neurosci. Lett..

[19] Kemmel V., Miehe M., Roussel G., Taleb O., Nail-Boucherie K., Marchand C., Stutz C., Andriamampandry C., Aunis D., Maitre M. (2006). Immunohistochemical Localization of a GHB Receptor-like Protein Isolated from Rat Brain. J. Comp. Neurol..

[20] Bay T., Eghorn L.F., Klein A.B., Wellendorph P. (2014). GHB Receptor Targets in the CNS: Focus on High-Affinity Binding Sites. Biochem. Pharmacol..

[21] Kaupmann K., Cryan J.F., Wellendorph P., Mombereau C., Sansig G., Klebs K., Schmutz M., Froestl W., van der Putten H., Mosbacher J. (2003). Specific Gamma-Hydroxybutyrate-Binding Sites but Loss of Pharmacological Effects of Gamma-Hydroxybutyrate in GABA(B)(1)-Deficient Mice. Eur. J. Neurosci..

[22] Schuler V., Lüscher C., Blanchet C., Klix N., Sansig G., Klebs K., Schmutz M., Heid J., Gentry C., Urban L. (2001). Epilepsy, Hyperalgesia, Impaired Memory, and Loss of Pre- and Postsynaptic GABA(B) Responses in Mice Lacking GABA(B(1)). Neuron.

[23] Ossato A., Vigolo A., Trapella C., Seri C., Rimondo C., Serpelloni G., Marti M. (2015). JWH-018 Impairs Sensorimotor Functions in Mice. Neuroscience.

[24] Maitre M., Andriamampandry C., Kemmel V., Schmidt C., Hodé Y., Hechler V., Gobaille S. (2000). Gamma-Hydroxybutyric Acid as a Signaling Molecule in Brain. Alcohol.

[25] Borgen L.A., Okerholm R., Morrison D., Lai A. (2003). The Influence of Gender and Food on the Pharmacokinetics of Sodium Oxybate Oral Solution in Healthy Subjects. J. Clin. Pharmacol..

[26] Elliott S., Lowe P., Symonds A. (2004). The Possible Influence of Micro-Organisms and Putrefaction in the Production of GHB in Post-Mortem Biological Fluid. Forensic Sci. Int..

[27] Aizawa M., Ito Y., Fukuda H. (1997). Roles of Gamma-Aminobutyric AcidB (GABA B) and Gamma-Hydroxybutyric Acid Receptors in Hippocampal Long-Term Potentiation and Pathogenesis of Absence Seizures. Biol. Pharm. Bull..

[28] Farr S.A., Uezu K., Creonte T.A., Flood J.F., Morley J.E. (2000). Modulation of Memory Processing in the Cingulate Cortex of Mice. Pharmacol. Biochem. Behav..

[29] Arfè R., Bilel S., Tirri M., Frisoni P., Serpelloni G., Neri M., Boccuto F., Bernardi T., Foti F., De-Giorgio F. (2021). Comparison of N-Methyl-2-Pyrrolidone (NMP) and the “Date Rape” Drug GHB: Behavioral Toxicology in the Mouse Model. Psychopharmacology.

[30] Frisoni P., Neri M., D’Errico S., Alfieri L., Bonuccelli D., Cingolani M., Di Paolo M., Gaudio R.M., Lestani M., Marti M. (2022). Cytokine Storm and Histopathological Findings in 60 Cases of COVID-19-Related Death: From Viral Load Research to Immunohistochemical Quantification of Major Players IL-1β, IL-6, IL-15 and TNF-α. Forensic Sci. Med. Pathol..

